# Femtosecond Laser Patterning of the Biopolymer Chitosan for Biofilm Formation

**DOI:** 10.3390/ijms17081243

**Published:** 2016-08-19

**Authors:** Regina Estevam-Alves, Paulo Henrique Dias Ferreira, Andrey C. Coatrini, Osvaldo N. Oliveira, Carla Raquel Fontana, Cleber Renato Mendonca

**Affiliations:** 1 São Carlos Institute of Physics, University of São Paulo (USP), São Carlos 13566-590, SP, Brazil; estevam.regina@gmail.com (R.E.-A.); andreycoatrini@gmail.com (A.C.C.); chu@ifsc.usp.br (O.N.O.J.); 2 Department of Materials Engineering, School of Engineering of São Carlos (USP), São Carlos 13563-120, SP, Brazil; 3 Physics Department, Federal University of São Carlos (UFSCAR), São Carlos 13565-905, SP, Brazil; paulohdf@df.ufscar.br; 4 Faculdade de Ciencias Farmaceuticas, UNESP—Univ. Estadual Paulista, Campus Araraquara, Departamento de Analises Clinicas, Araraquara 14800-903, SP, Brazil

**Keywords:** fs-laser micromachining, micropatterning, chitosan, bacterial growth

## Abstract

Controlling microbial growth is crucial for many biomedical, pharmaceutical and food industry applications. In this paper, we used a femtosecond laser to microstructure the surface of chitosan, a biocompatible polymer that has been explored for applications ranging from antimicrobial action to drug delivery. The influence of energy density on the features produced on chitosan was investigated by optical and atomic force microscopies. An increase in the hydrophilic character of the chitosan surface was attained upon laser micromachining. Patterned chitosan films were used to observe *Staphylococcus aureus* (ATCC 25923) biofilm formation, revealing an increase in the biofilm formation in the structured regions. Our results indicate that fs-laser micromachining is an attractive option to pattern biocompatible surfaces, and to investigate basic aspects of the relationship between surface topography and bacterial adhesion.

## 1. Introduction

Nano- and micro-patterned surfaces have attracted increasing attention in recent years among the scientific communities of physics, chemistry, medicine and biology [1,2,3,4]. In particular, it has been shown that the surface chemistry and topography (surface structure) of biomaterial substrates has a strong effect not only on cell morphology [5], but it can also have an influence on regulating cell behavior, such as adhesion, migration, orientation, guidance, differentiation, proliferation, gene expression and protein synthesis [6]. Structured surfaces are now used in biosensors, in biochips for diagnostics and cell microarrays, in drug delivery and in prostheses for medical implants. Understanding cell-substrate interactions is crucial to further develop these technologies efficiently [7]. Surface adhesion, for instance, is known as a survival mechanism for bacteria [8]. Therefore, the understanding of the bacterial-surface interaction mechanism is essential for the design of biomaterial surfaces with improved properties, to either allow or prevent bacteria attachment. The interactions between bacterial cells and different material surfaces such as glass, ceramics, metals and polymers have been studied to minimize or prevent bacterial colonization [9,10,11,12]. Cell adhesion is governed by several factors, including the physicochemical properties of the cell and of the substrate, and the conditions under which attachment takes place [13]. Bacterial adhesion, in particular, is less understood because of the diversity and complexity of both the bacterial cells and the surfaces [11]. Basically, two types of strategies to control cell adhesion have been used: (i) chemical or physical modification of substrates and (ii) coating the surface with biocompatible and/or bioactive agents favorable for cell attachment [11].

Surfaces have been modified by coating with nanostructured films or by changes in topography [14,15], in some cases with decreased bio-adhesion by tailored micropatterns [16], and in others to allow control of the cell growth direction [17]. Another approach to achieve controlled surface textures is femtosecond laser micromachining [18,19], which is advantageous in comparison to similar methods because it involves a direct, single-step and maskless procedure [20,21,22,23]. Although most of the papers using surface modification by laser technologies are focused on studying cell behavior, there are only a few studies aimed at bacterial growth.

In this paper we study the fs-laser microstructuring on films of the biopolymer chitosan, a linear cationic polysaccharide [(1→4)-2-amino-2-deoxy-β-d-glucopyranose] obtained from the deacetylation of chitin [(1→4)-2-acetamido-2-deoxy-β-d-glucopyranose] encountered in crustaceans. Chitosan is biocompatible and biodegradable, and is explored as an antimicrobial agent in blood coagulation, taste sensors, bone regeneration, controlled drug delivery and conductive membranes [24,25,26,27,28,29,30,31]. We studied the influence of energy density on chitosan micromachining, the features of which were characterized by optical and atomic force microscopy. We were able to determine the energy density threshold for biopolymer removal, distinguishing it from the energy density range that leads to changes in the material. The wetting properties of micromachined surfaces were characterized by water contact angle measurements, which revealed an increase in the hydrophilic character of the chitosan surface. Once the proper fs-laser micromachining conditions were determined, we patterned chitosan films that were used as templates to observe *Staphylococcus aureus* biofilm formation. The results obtained indicate that micropatterned surfaces increase the hydrophilic character of the chitosan sample, which helps biofilm formation. Overall, we provide evidence that fs-laser micromachining is adequate to pattern biocompatible surfaces, with which one can also investigate the dependence of surface topography on bacterial adhesion.

## 2. Results and Discussion

**Figure 1:**
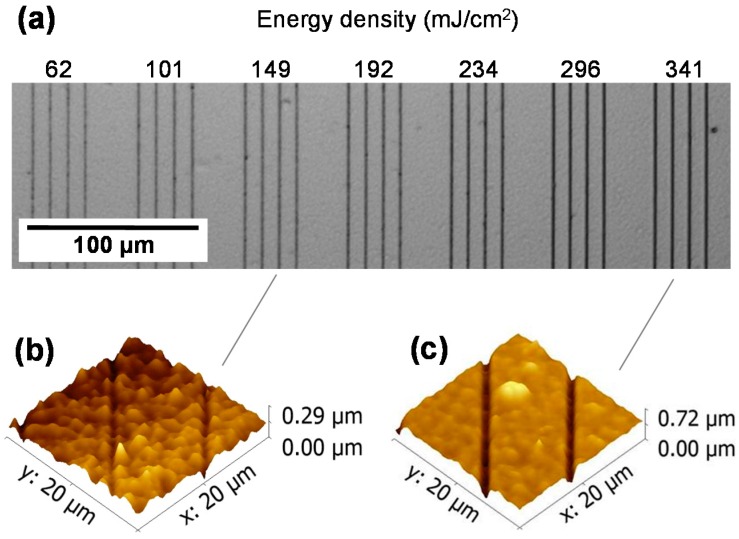
(**a**) Transmission optical microcopy of lines micromachined in chitosan film at a translation speed of 50 μm/s and distinct energy densities. Three-dimensional AFM images of micromachined chitosan surface with energy densities of (**b**) 149 and (**c**) 341 mJ/cm^2^.

The influence of energy density on fs-laser micromachining features was analyzed by optical and atomic force microscopy. Figure 1a shows an optical microscopy image of microstructures produced in chitosan at a translation speed of 50 μm/s. Line patterns (500 μm long separated by 10 μm) were directly micromachined on the film surface using different energy densities (from 62 to 341 mJ/cm^2^). Figure 1b,c show two three-dimensional atomic force microscopy (AFM) morphological images of the micromachined region at energy densities of 149 and 341 mJ/cm^2^, respectively. When energy densities above 101 mJ/cm^2^ are used, the removal of the chitosan from the sample is observed, as illustrated in the AFM images. For energy densities below 62 mJ/cm^2^, no removal of material was observed from the sample surface (data not shown), indicating that the energy density threshold for chitosan ablation is at about 62 mJ/cm^2^ [32,33]. Therefore, the micromachined lines observed in Figure 1a for an energy density of 62 mJ/cm^2^ are only related to material changes and not removal.

**Figure 2:**
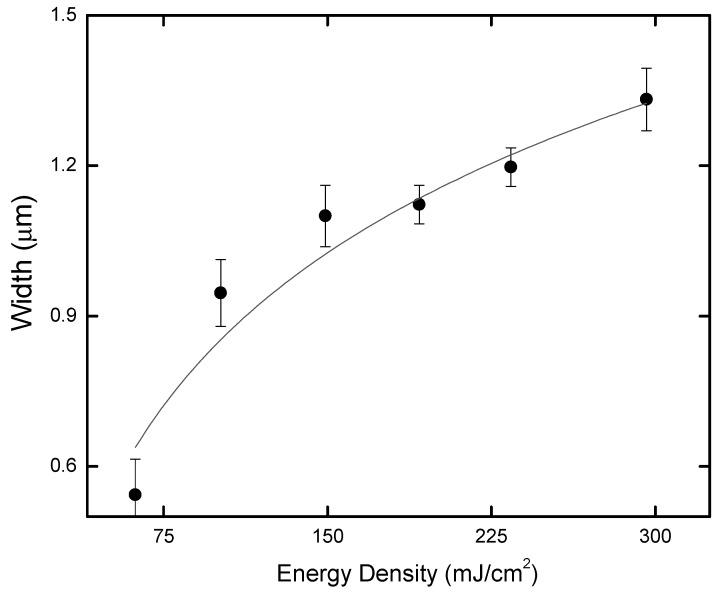
Width of micromachined lines as a function of energy density, for a scan speed of 50 μm/s. The solid line is only drawn to guide the eye.

The width of the lines fabricated on the chitosan film increases with the energy density, as illustrated in Figure 2. As can be seen, the obtained groove width ranges from (0.54 ± 0.07) μm up to (1.33 ± 0.06) µm, representing an increase of approximately 2.5 when the energy density is changed from about 60 to 300 mJ/cm^2^.

**Figure 3:**
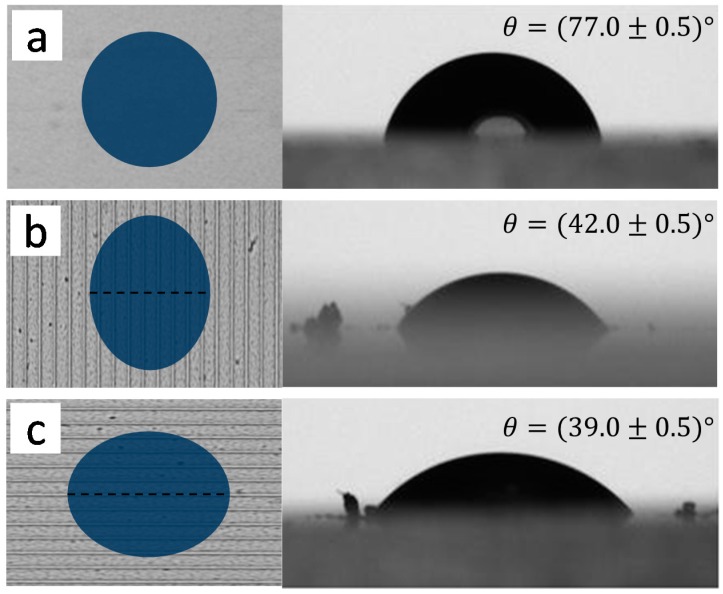
Water contact angle measured for: (**a**) reference sample (*θ* = (77.0 ± 0.5)°); (**b**) perpendicular to the microstrucutred lines (*θ* = (42.0 ± 0.5)°); (**c**) parallel to the microstructured lines (*θ* = (39.0 ± 0.5)°). The dashed lines represent the direction along which the contact angles were obtained. The left column is only an illustration of the contact angle anisotropy observed in the right column.

The use of biomaterials for applications in tissue engineering and implants depends on their surface characteristics, mainly the degree of hydrophobicity or hydrophilicity that has been reported to be an important factor influencing cell adhesion [34,35]. Microstructured chitosan surfaces revealed interesting wetting characteristics, as can be noted in Figure 3, for images and contact angles obtained with the sessile drop method on micromachined surfaces (grooves) composed of 3-mm-long lines separated by 10 μm (total area of 9.0 mm^2^).

The contact angles were (77.0 ± 0.5)° for the non-microstructured surface (reference) and in the order of 40° for the structured surfaces made with 487 mJ/cm^2^ energy density. Also, the contact angle on the structured surface is anisotropic, for it depends on the observation direction of the produced pattern (lines). As illustrated in Figure 3b,c, the contact angle was (42.0 ± 0.5)° and (39.0 ± 0.5)° when observed perpendicularly and parallel to the microstructured lines, respectively. The dashed lines in Figure 3b,c represent the direction along which the images for the contact angle measurements were obtained. Thus, surface roughness caused by the fs-laser microstructuring increases the hydrophilic character of the original chitosan sample, a behavior that can be explained by the Wenzel model [36], which assumes a homogeneous contact between the water droplet and the polymer surface.

**Figure 4:**
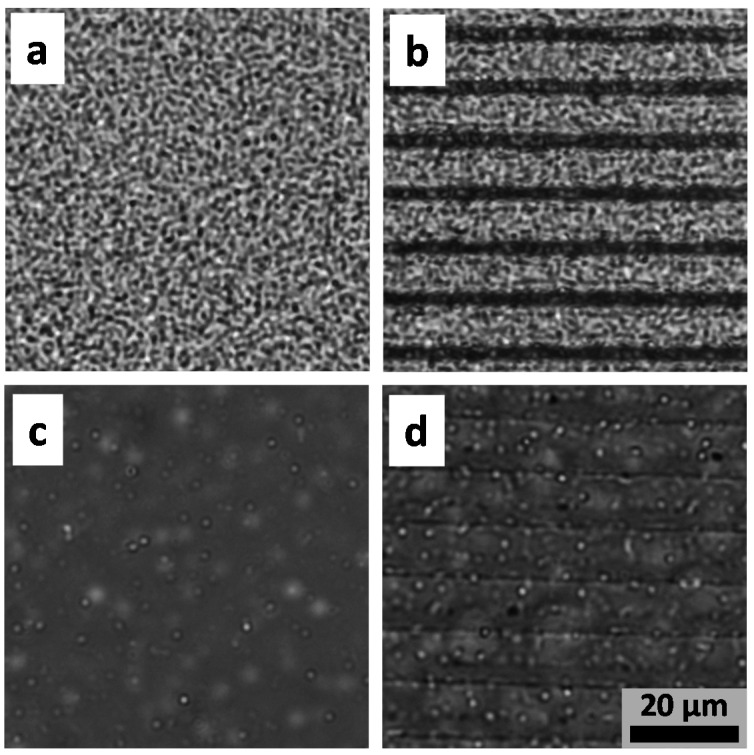
Optical microscope images of microenvironments with biofilm formation on the unpatterned (**a**) and micropatterned (**b**) PMMA surfaces; and unpatterned (**c**) and micropatterned (**d**) chitosan surfaces.

Wetting characteristics can affect microbial interactions and, hence, bio-adhesion control. To demonstrate the potential of the micromachined surfaces to allow for the adhesion of bacteria, we used a 162 mJ/cm^2^ energy density at a 50 μm/s scan speed to fabricate micropatterned surfaces on chitosan films (thickness of approximately 3 μm). These parameters were found to be adequate for micropatterning according to Figure 2. Each microenvironment was composed by 500-μm-long lines separated by distinct spacing, varying from 4 to 12 μm. The total area of each microenvironment was 500 × 500 μm^2^. For comparison, the same microenvironments were fabricated on 430-μm-thick poly(methyl methacrylate) (PMMA) films. Figure 4 shows optical microscopy images of bacteria grown on micropatterned surfaces (lines spaced by 10 μm). Similar results were observed for the other spacing used. For the control PMMA sample, no difference was observed on the *Staphylococcus aureus* grown on the unpatterned (Figure 4a) and micropatterned surface (Figure 4b), i.e., the density of bacteria between the microfabricated lines is the same as the one observed on the unpatterned surface. For the chitosan substrate, however, the images in Figure 4c (unpatterned surface) and Figure 4d (10 μm patterned surface) reveal an increased bacteria density, seen as white dots (focus) and cloudy spots (out-of-focus), on the structured sample. According to [37], the effect of topography on cell adhesion is associated with the creation of a stable hydrophobic state, in which air pockets at the microscopic features inhibit the interaction of microbes with the surface. As displayed in the results of Figure 3, the microstructured pattern on chitosan films creates a more hydrophilic surface, which helps the interaction with bacteria cells, enabling adhesion and, therefore, biofilm formation. The overall smaller bacterial density on the chitosan substrate in comparison to that on PMMA samples is explained by the well-known antimicrobial action of chitosan [29,38]. For PMMA, given the high bacterial density achieved, the effect of the surface microstructuring is not evidenced.

**Figure 5:**
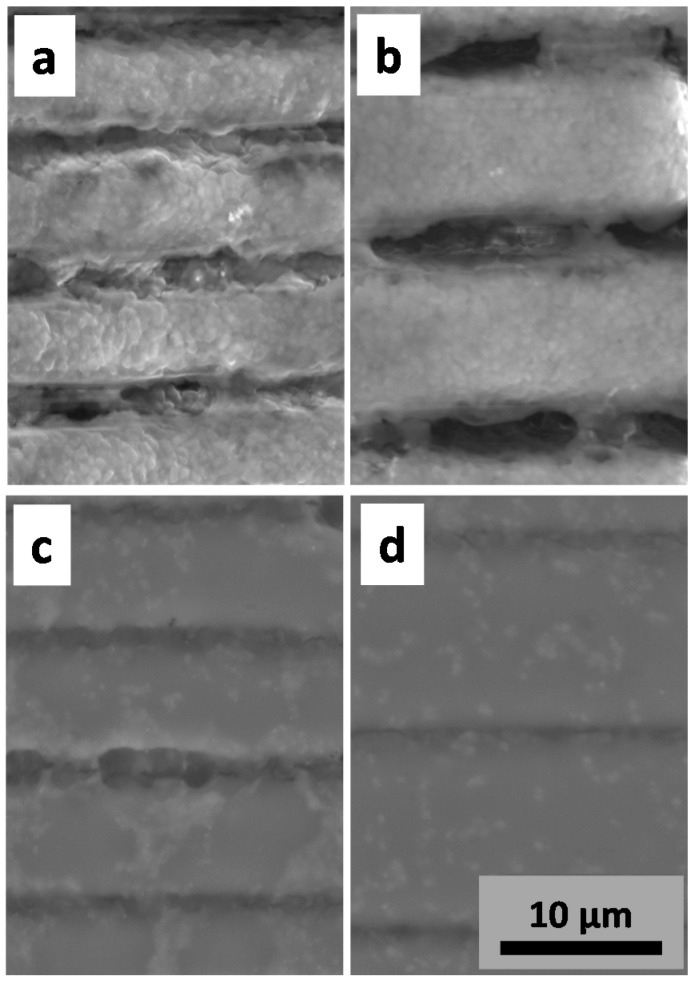
SEM images of microenvironments with biofilm formation on the 8 μm (**a**) and 12 μm (**b**) micropatterned PMMA surface; and 8 μm (**c**) and 12 μm (**d**) micropatterned chitosan surface.

Biofilm formation initiates with microbial adhesion to biotic or abiotic surfaces. Growth of adherent organisms results in cluster formation referred to as complex aggregates or microcolonies. Figure 5 shows SEM images of biofilm formation on PMMA and chitosan surfaces patterned with lines separated by 8 and 12 μm. The PMMA microstructured surfaces (Figure 5a,b) exhibit dense biofilm after five days, as compared to chitosan (Figure 5c,d), which is related to the antimicrobial characteristic of chitosan. Also, the results show a higher density of bacteria in the grooves microstructured with 8 μm (Figure 5a,c) in comparison to the ones at 12 μm (Figure 5b,d), respectively. Such results indicate that the closer the microstructured lines are, the greater the bacterial interaction, because nucleation and colonization are favored by niches created by fs-laser micromachining.

Owing to the antimicrobial chitosan activity, the chitosan samples (Figure 5c,d) were shown to resist biofilm formation, inhibiting bacterial attachment. However, even with the antimicrobial effect of chitosan, a behavior similar to the one displayed for PMMA samples was observed; as lines became closer, a denser biofilm was obtained on the chitosan microstructured surface, indicating an increase in bio-adhesion. Therefore, surface microstructuring can contribute to nucleation and guide the formation of three-dimensional mature biofilms. For separation between lines in the microstructured surfaces larger than 12 μm, no difference was observed on the biofilm as compared to the unpatterned surface (data not shown).

In summary, there is a tradeoff between the natural antibacterial action from chitosan and the increasing adhesion of the microstructured surfaces, which promotes biofilm formation. Furthermore, micropatterning only affects bacterial growth if the grooves created by fs-laser micromachining are smaller than a given size, since 12 μm microstructured surfaces showed no difference compared to the smooth surface.

## 3. Materials and Methods

Chitosan was purchased from Galena Chemistry & Pharmaceutical (Campinas, Brazil) and used as received. It was dissolved in acetic acid (4 mg/mL) and deposited onto glass substrates by spin coating to form ca. 100-nm-thick films. For the bacterial growth experiments, cast chitosan films with thickness up to approximately 3 μm were prepared with (4% *w*/*v*) solutions being spread onto glass substrates (1.5 mm diameter). The chitosan films were micromachined using an extended-cavity Ti:Sapphire laser oscillator, centered at 800 nm and operating at a repetition rate of 5.2 MHz, which produces pulses with energy up to 100 nJ and duration of 50 fs. The beam was focused on the sample surface by a 40× (0.67-NA) microscope objective. Further details about the micromachining system can be found elsewhere [39]. The sample was moved at a constant speed with respect to the laser beam using a computer-controlled xyz stage. Sample morphology was analyzed using an Atomic Force Microscope (AFM) from Nanosurf (Nanosurfe EasyScan 2 FlexAFM, Liestal, Switzerland) in the tapping mode, with images collected with high resolution (512 lines/scan) at a scan rate of 0.5 Hz. The samples were also examined with scanning electron microscopy (SEM), using a HITACHI TM 3000 microscope operating at 15 kV, and by optical microscopy using a LSM 700 from Zeiss (Jena, Germany). The wetting properties of the samples were studied by measuring the static contact angle (CA) for water, using a goniometer coupled to a horizontal microscope (KSV CAM 200, KSV, Helsinki, Finland). The CA measurements were performed at 25 °C and relative air humidity around 40%. Water droplets with a volume of about 3 μL (radius of ca. 1 mm) were used for all measurements.

For the biological studies we used the American Type Culture Collection (Manassas, VA, USA) reference strain of *Staphylococcus aureus* (ATCC 25923), whose culture was maintained by weekly subculture in plates composed of Trypticase soy agar (Becton, Dickinson, and Co., Sparks, MD, USA). Biofilm development was performed by using the approach described in Reference [40]; 600 μL of the inoculum (~10^8^ cells/mL—estimated by spectrophotometry) were carefully pipetted to cover the samples (3-μm thick microstructures chitosan substrates) placed in each well of 6-well plates. The plates were then incubated aerobically at 35 °C for five days. After an initial incubation period of 48 h, the liquid medium was carefully aspirated from each well and the biofilms were replenished with fresh broth. Then, fresh Tryptic Soy Broth (TSB) was added daily into each well, very slowly to avoid disrupting the biofilm. The chitosan films coated with biofilms were analyzed with SEM, for which the samples were thoroughly washed with sterile 0.1 M phosphate buffer (pH 7.4). The washing procedure was repeated three times and 1mL of 3% glutaraldehyde and 2% paraformaldehyde in 0.1 M potassium phosphate buffer, pH 7.4, was added. Then, three washes with pure buffer solution were performed. Dehydration was carried out with increasing concentrations of ethanol (50%, 60%, 70%, 80%, 90% and 100%). Following dehydration, the samples were dried in a desiccator with silica for 72 h and then analyzed in the microscope.

## 4. Conclusions

This paper focused on the fs-laser microfabrication of a chitosan surface and its characterization, aiming at the production of engineered surface topographies to control bio-adhesion. Micropatterned chitosan surfaces are more hydrophilic than a smooth chitosan film, which helps the formation of *Staphylococcus aureus* biofilms. Our results also indicate a balance between increased adhesion on microstructured surfaces and the natural antibacterial action of chitosan. The results of this study demonstrated that fs-laser micromachining is an interesting option to pattern bio-surfaces to study bacterial growth and development, which can be used to elucidate the connection between surface topography and bacterial adhesion. The applicability of the methodology can even be extended if modified chitosans are employed, e.g., with antibiotic-loaded chitosan gels [41].

## References

[1] Caruso F. (2001). Nanoengineering of particle surfaces. Adv. Mater..

[2] Cheng J.-Y., Wei C.-W., Hsu K.-H., Young T.-H. (2004). Direct-write laser micromachining and universal surface modification of PMMA for device development. Sens. Actuators B Chem..

[3] Katsikogianni M.G., Missirlis Y.F. (2010). Bacterial adhesion onto materials with specific surface chemistries under flow conditions. J. Mater. Sci. Mater. Med..

[4] Bazaka K., Jacob M.V., Crawford R.J., Ivanova E.P. (2011). Plasma-assisted surface modification of organic biopolymers to prevent bacterial attachment. Acta Biomater..

[5] Liu Y., Sun S., Singha S., Cho M.R., Gordon R.J. (2005). 3D femtosecond laser patterning of collagen for directed cell attachment. Biomaterials.

[6] Murugan R., Molnar P., Rao K.P., Hickman J.J. (2009). Biomaterial surface patterning of self-assembled monolayers for controlling neuronal cell behaviour. Int. J. Biomed. Eng. Technol..

[7] Anselme K., Davidson P., Popa A.M., Giazzon M., Liley M., Ploux L. (2010). The interaction of cells and bacteria with surfaces structured at the nanometre scale. Acta Biomater..

[8] Busscher H.J., van der Mei H.C. (2012). How do bacteria know they are on a surface and regulate their response to an adhering state?. PLoS Pathog..

[9] Mitik-Dineva N., Wang J., Truong V., Stoddart P., Malherbe F., Crawford R., Ivanova E. (2009). *Escherichia coli*, *Pseudomonas aeruginosa*, and *Staphylococcus aureus* Attachment Patterns on Glass Surfaces with Nanoscale Roughness. Curr. Microbiol..

[10] Truong V., Rundell S., Lapovok R., Estrin Y., Wang J., Berndt C., Barnes D., Fluke C., Crawford R., Ivanova E. (2009). Effect of ultrafine-grained titanium surfaces on adhesion of bacteria. Appl. Microbiol. Biotechnol..

[11] Ivanova E.P., Mitik-Dineva N., Wang J., Pham D.K., Wright J.P., Nicolau D.V., Mocanasu R.C., Crawford R.J. (2008). Staleya guttiformis attachment on poly(tert-butylmethacrylate) polymeric surfaces. Micron.

[12] Puckett S.D., Taylor E., Raimondo T., Webster T.J. (2010). The relationship between the nanostructure of titanium surfaces and bacterial attachment. Biomaterials.

[13] Crawford R.J., Webb H.K., Truong V.K., Hasan J., Ivanova E.P. (2012). Surface topographical factors influencing bacterial attachment. Adv. Colloid Interface Sci..

[14] Cooke M.J., Phillips S.R., Shah D.S.H., Athey D., Lakey J.H., Przyborski S.A. (2008). Enhanced cell attachment using a novel cell culture surface presenting functional domains from extracellular matrix proteins. Cytotechnology.

[15] Alves N.M., Shi J., Oramas E., Santos J.L., Tomas H., Mano J.F. (2009). Bioinspired superhydrophobic poly(l-lactic acid) surfaces control bone marrow derived cells adhesion and proliferation. J. Biomed. Mater. Res. A.

[16] Kim E., Kinney W.H., Ovrutsky A.R., Vo D., Bai X., Honda J.R., Marx G., Peck E., Lindberg L., Falkinham J.O. (2014). A surface with a biomimetic micropattern reduces colonization of *Mycobacterium abscessus*. FEMS Microbiol. Lett..

[17] Langheinrich D., Yslas E., Broglia A., Rivarola V., Acevedo D., Lasagni A. (2012). Control of cell growth direction by direct fabrication of periodic micro- and submicrometer arrays on polymers. J. Polym. Sci. B Polym. Phys..

[18] Nayak B.K., Kolasinski K.W. (2008). Formation of nano-textured conical microstructures in titanium metal surface by femtosecond laser irradiation. Appl. Phys..

[19] Ortiz R., Moreno-Flores S., Quintana I., Vivanco M., Sarasua J.R., Toca-Herrera J.L. (2014). Ultra-fast laser microprocessing of medical polymers for cell engineering applications. Mater. Sci. Eng. C.

[20] Davis K.M., Miura K., Sugimoto N., Hirao K. (1996). Writing waveguides in glass with a femtosecond laser. Opt. Lett..

[21] Eldada L., Shacklette L.W. (2000). Advances in polymer integrated optics. IEEE J. Sel. Top. Quantum Electron..

[22] Cumpston B.H., Ananthavel S.P., Barlow S., Dyer D.L., Ehrlich J.E., Erskine L.L., Heikal A.A., Kuebler S.M., Lee I.Y.S., McCord-Maughon D. (1999). Two-photon polymerization initiators for three-dimensional optical data storage and microfabrication. Nature.

[23] Maruo S., Nakamura O., Kawata S. (1997). Three-dimensional microfabrication with two-photon-absorbed photopolymerization. Opt. Lett..

[24] He Q., Gong K., Ao Q., Ma T., Yan Y., Gong Y., Zhang X. (2011). Positive charge of chitosan retards blood coagulation on chitosan films. J. Biomater. Appl..

[25] Dos Santos D.S., Riul A., Malmegrim R.R., Fonseca F.J., Oliveira O.N., Mattoso L.H.C. (2003). A layer-by-layer film of chitosan in a taste sensor application. Macromol. Biosci..

[26] Bojar W., Kucharska M., Ciach T., Koperski L., Jastrzębski Z., Szałwiński M. (2014). Bone regeneration potential of the new chitosan-based alloplastic biomaterial. J. Biomater. Appl..

[27] Bhattarai N., Gunn J., Zhang M. (2010). Chitosan-based hydrogels for controlled, localized drug delivery. Adv. Drug Deliv. Rev..

[28] Xiong Y., Wang H., Wu C., Wang R. (2012). Preparation and characterization of conductive chitosan-ionic liquid composite membranes. Polym. Adv. Technol..

[29] Rabea E.I., Badawy M.E.-T., Stevens C.V., Smagghe G., Steurbaut W. (2003). Chitosan as antimicrobial agent: Applications and mode of action. Biomacromolecules.

[30] Hu Q., Li B., Wang M., Jiacong Shen J. (2004). Preparation and characterization of biodegradable chitosan/hydroxyapatite nanocomposite rods via in situ hybridization: A potential material as internal fixation of bone fracture. Biomaterials.

[31] Liang D., Lu Z., Yang H., Gao J., Chen R. (2016). Novel Asymmetric Wettable AgNPs/Chitosan Wound Dressing: In Vitro and In Vivo Evaluation. ACS Appl. Mater. Interface.

[32] Estevam-Alves R., Ferreira P.H.D., Almeida G.F.B., Sousa W.S., Mendonça C.R. (2014). Microfabrication of electroluminescent polymer for devices construction. Appl. Surf. Sci..

[33] Correa D.S., Cardoso M.R., Tribuzi V., Misoguti L., Mendonca C.R. (2012). Femtosecond Laser in Polymeric Materials: Microfabrication of Doped Structures and Micromachining. IEEE J. Sel. Top. Quantum Electron..

[34] Dulinska-Molak I., Lekka M., Kurzydłowski K.J. (2013). Surface properties of polyurethane composites for biomedical applications. Appl. Surf. Sci..

[35] Tang Z., Akiyama Y., Okano T. (2012). Temperature-responsive polymer modified surface for cellsheet engineering. Polymers.

[36] Wenzel R.N. (1936). Resistance of solid surfaces to wetting by water. Ind. Eng. Chem..

[37] Decker J.T., Kirschner C.M., Long C.J., Finlay J.A., Callow M.E., Callow J.A., Brennan A.B. (2013). Engineered Antifouling Microtopographies: An Energetic Model That Predicts Cell Attachment. Langmuir.

[38] Regiel-Futyra A., Kus-Liśkiewicz M., Sebastian V., Irusta S., Arruebo A., Stochel G., Kyzioł A. (2015). Development of Noncytotoxic Chitosan–Gold Nanocomposites as Efficient Antibacterial Materials. ACS Appl. Mater. Interface.

[39] Ferreira P.H.D., Stefanutti R., Pavinatto F.J., Mendonça C.R. (2014). Femtosecond laser fabrication of waveguides in DR13-doped PMMA. Opt. Commun..

[40] Fontana C.R., Abernethy A.D., Som S., Ruggiero K., Doucette S., Marcantonio R.C., Boussios C.I., Kent R., Goodson J.M., Tanner A.C.R., Soukos N.S. (2009). The antibacterial effect of photodynamic therapy in dental plaque-derived biofilms. J. Periodontal Res..

[41] Wu F., Meng G., He J., Wu Y., Wu F., Gu Z. (2014). Antibiotic-Loaded Chitosan Hydrogel with Superior Dual Functions: Antibacterial Efficacy and Osteoblastic Cell Responses. ACS Appl. Mater. Interface.

